# Topographical Relationship Between Acute Macular Neuroretinopathy and Choroidal Watershed Zone or Patchy Choroidal Filling

**DOI:** 10.3389/fmed.2022.762609

**Published:** 2022-02-01

**Authors:** Jialiang Duan, Jianbin An, Minhao Li, Zhengwei Zhang, Liang Zhou, Pengfei Yin, Jingxue Ma, Qingli Shang

**Affiliations:** ^1^Department of Ophthalmology, The Second Hospital of Hebei Medical University, Shijiazhuang, China; ^2^Department of Ophthalmology, The Affiliated Wuxi No. 2 People's Hospital of Nanjing Medical University, Wuxi, China; ^3^Department of Ophthalmology, Second Xiangya Hospital, Central South University, Changsha, China; ^4^Department of Ophthalmology, The Third Affiliated Hospital of Hebei Medical University, Shijiazhuang, China

**Keywords:** acute macular neuroretinopathy (AMN), choroidal watershed zone, indocyanine green angiography (ICGA), near-infrared reflectance imaging, patchy choroidal filling

## Abstract

**Purpose:**

To study the topographical relationship between acute macular neuroretinopathy (AMN) lesions and the choroidal watershed zone (CWZ) or patchy choroidal filling (PCF) using multimodal imaging.

**Methods:**

Lesions in patients diagnosed with AMN were clinically examined using multimodal imaging, including fundus photography, near-infrared reflectance imaging, spectral-domain optical coherence tomography (OCT), fluorescein angiography, indocyanine green angiography, OCT angiography, and microperimetry. The topographical relationship between AMN and the CWZ or PCF was evaluated.

**Results:**

Seven eyes of six patients were included in the study. The mean age of the patients was 35.8 ± 11.7 years. The AMN lesions were collocated with the CWZ in five eyes and the PCF in one eye. Among these eyes, three had complete patterns, and three had partial patterns. Only one eye showed no topographical relationship between AMN and the CWZ or PCF.

**Conclusion:**

The colocation of AMN and CWZ/PCF suggests that the AMN lesions were within an area with a dual-watershed zone: the watershed zone between the retinal deep capillary plexus and choriocapillaris, and the choroidal watershed zone or patchy choroidal filling. This retinal area was highly vulnerable to hypoperfusion. Our results suggest a novel pathophysiological mechanism for AMN.

## Introduction

In 1975, Bos and Deutman (1) first described four cases of paracentral scotomas and dark-reddish lesions pointing to the fovea; they named this disorder “acute macular neuroretinopathy (AMN).” With the introduction of optical coherence tomography (OCT), reports of AMN have increased within the last decade (2). AMN is localized at the outer nuclear layer (ONL) and the outer plexiform layer (OPL) on OCT (3), but its underlying pathology is not completely understood. A vascular ischemic etiology has been proposed (3). The OPL and ONL are within the functional watershed zone between two sources of blood supply to the retina and choroid, and retinal ischemia or choroidal ischemia have been hypothesized as specific etiologies. However, each hypothesis on its own cannot fully explain the clinical findings of AMN, and the original location of the vascular impairment of AMN is still debated.

The choroidal watershed zone (CWZ) and patchy choroidal filling (PCF), which have poor vascularity, play a pathological role in several diseases, such as age-related macular degeneration (AMD) and choroidal neovascularization (CNV), because of their relatively low blood flow (4–6). The relationship between these areas and AMN has not been studied before.

In this study, we investigated the topographical relationships of the CWZ and PCF with AMN lesions using multi-modular imaging. Our results suggest a novel pathophysiological mechanism for AMN.

## Methods

This retrospective study included seven eyes of six patients from the Second Hospital of Hebei Medical University who reported between 2017 and 2021. The study adhered to the tenets of the Declaration of Helsinki, and the protocols were approved by the Ethical Committee of The Second Hospital of Hebei Medical University.

AMN was diagnosed based on the following criteria (2): (a) history of acute-onset paracentral or central scotoma, (b) parafoveal hyporeflective lesions on near-infrared reflectance imaging (NIR), and (c) hyperreflectivity of the OPL/ONL junction with associated attenuation of the underlying ellipsoid zone on spectral-domain OCT (SD-OCT). The collected data included age, sex, medical history, best-corrected visual acuity (BCVA), and slit-lamp findings. Fundus photography was performed with a retinal camera (Kowa Company, Nagoya, Japan). Fluorescein angiography (FA) and indocyanine green angiography (ICGA) were performed using confocal scanning laser ophthalmoscope angiography (Heidelberg Engineering, Heidelberg, Germany). For the early stage, the images were obtained immediately after injecting fluorescein and indocyanine green dye. CWZ and PCF were evaluated mainly using ICGA, as the normal choroidal vasculature is usually difficult to assess using FA. CWZ was characterized by vertical, angled, or stellate-shaped zones of transient choroidal hypofluorescence during the early phase of ICGA produced by the delayed filling of the choriocapillaris (6, 7). PCF, which is similar but different from the CWZ, was defined as an isolated area with, at least, half a disc diameter of transient choroidal hypofluorescence during the early phase of ICGA (5). A minimum of two clear transit frames of ICGA images were required to evaluate the CWZ and PCF. The eyes with AMN that had not undergone ICGA examination, had no early-phase ICGA images, or had poor-quality early-phase ICGA images were excluded from our study. The extent of AMN was determined using NIR. NIR and SD-OCT were performed using Spectralis HRA OCT (Heidelberg Engineering, Heidelberg, Germany). Microperimetry was performed using an MP-3 microperimeter (Nidek, Aichi, Japan). OCT angiography (OCT-A) was performed using AngioPlex Cirrus 5000 HD-OCT (Carl Zeiss Meditec, Inc., Dublin, California, USA).

The topographical relationship of AMN with CWZ or PCF was determined using NIR and ICGA and analyzed by two readers (JD, ZZ). The patterns of colocation were classified into three groups: complete, partial, and none.

## Results

Six patients (seven eyes) were included in the study: two (33.3%) were female; the mean age was 35.8 ± 11.7; and two patients (33.3%) had bilateral involvement.

The colocation of AMN lesions and CWS/PCF was detected in six eyes (85.7%); three had complete patterns, and three had partial patterns. Only one case (Case 3) showed no topographical relationship between the AMN lesions and CWS/PCF. Table 1 summarizes the demographic, ocular, and systemic characteristics of the enrolled patients.

**Table 1:** Summary of patient demographics, ocular, and systemic findings.

**Case**	**Eye**	**Sex**	**Age**	**Systemic findings or PMH**	**Co-localization of AMN and WSZ/PCF**	**Baseline BCVA**	**Final BCVA**
1	OU (Only OS included in this study)	F	37	High-Myopia; Suspected of Behçet's disease	Complete	20/25	20/20
2	OS	M	33	LASIK surgery	Complete	20/30	20/20
3	OS	F	32	Flu-like prodrome	None	20/40	20/25
4	OS	M	56	Renal hamartomas underwent partial nephrotomy	Complete	20/40	20/30
5	OD	M	37	Mild myopia, HTN	Partial	20/40	20/25
	OS				Partial	20/30	20/25
6	OS	M	20	None	Partial	20/200	20/50

*AMN, Acute macular neuroretinopathy; BCVA, best corrected visual acuity; F, female; HTN, hypertension; M, male; PMH, Past medical history; WSZ, Watershed zone; PCF, patchy choroidal filling*.

Subsequently, we describe the representative cases individually.

### Case 1

**Figure 1:**
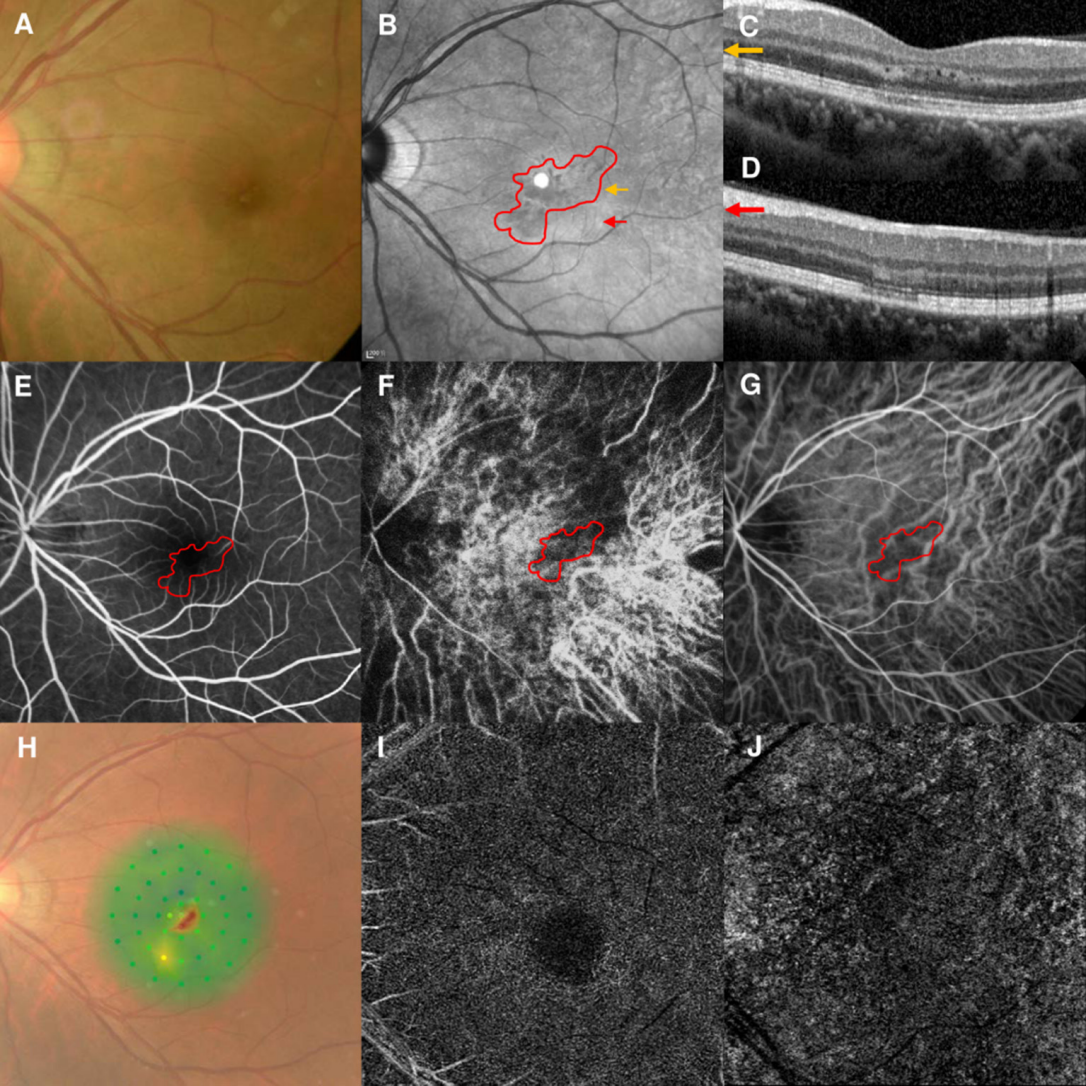
Case 1: Multimodal imaging of acute macular neuroretinopathy in the left eye. Fundus photography **(A)** and corresponding near-infrared reflectance imaging (NIR) demonstrated multiple hyporeflective lesions at the parafoveal region in the left eye [**(B)**, red line]. Spectral-domain optical coherence tomography (OCT) line scans [**(C,D)**, the corresponding areas are marked by the yellow arrow and red arrow, with the noted areas on NIR] showed irregular hyperreflectivity at the outer plexiform and outer nuclear layers. The findings during the arteriovenous phase of fluorescein angiography (FA) were unremarkable **(E)**. The early phase of indocyanine green angiography (ICGA) showed a stellate watershed zone and AMN lesions located in the watershed zone **(F)**; the watershed zone could not be observed after 4 s on ICGA imaging **(G)**. The corresponding hypofluorescent areas on NIR were identified and co-localized; these are marked by the red line, with the noted areas on FA and ICGA. Microperimetry showed a scotoma corresponding to the hyporeflective lesions on NIR **(H)**. OCT angiography showed normal vascular flow at the deep capillary plexus **(I)** and choriocapillaris **(J)**.

A 37-year-old woman with high myopia in both eyes had a 4-day history of bilateral blurred vision with a paracentral scotoma. Although she denied other illnesses, recent vaccinations, and any medication use, she was suspected of having Behçet disease due to recurrent mouth ulcers. The best-corrected visual acuities (BCVAs) were 20/25 OD and 20/40 OS. The fundus examination findings were unremarkable (Figure 1A). NIR of the left eye showed multiple irregular hyporeflective areas (Figure 1B), while the SD-OCT showed irregular hyperreflectivity of the OPL and ONL in the left eye (Figures 1C,D). The arteriovenous phase of the FA showed that the AMN lesions were located at the terminus of the perifoveal small vessels in the left eye (Figure 1E). Early-phase ICGA showed a stellate-patterned watershed zone across the macula, and the AMN lesions were located within the inferior part of the watershed zone (Figure 1F). This watershed zone could not be observed after 4 s (Figure 1G). Microperimetry revealed a scotoma corresponding to the hyporeflective lesions revealed by NIR (Figure 1H). OCT-A did not show vascular flow voids at the level of the deep capillary plexus (DCP; Figure 1I) or the choriocapillaris (CC; Figure 1J), and the OCT-A images were according to the myopic status of this patient. The right eye was not included in our study because we did not have an early-phase ICGA image available. The BCVAs improved to 20/20 OU after 9 months of follow-up.

### Case 2

**Figure 2:**
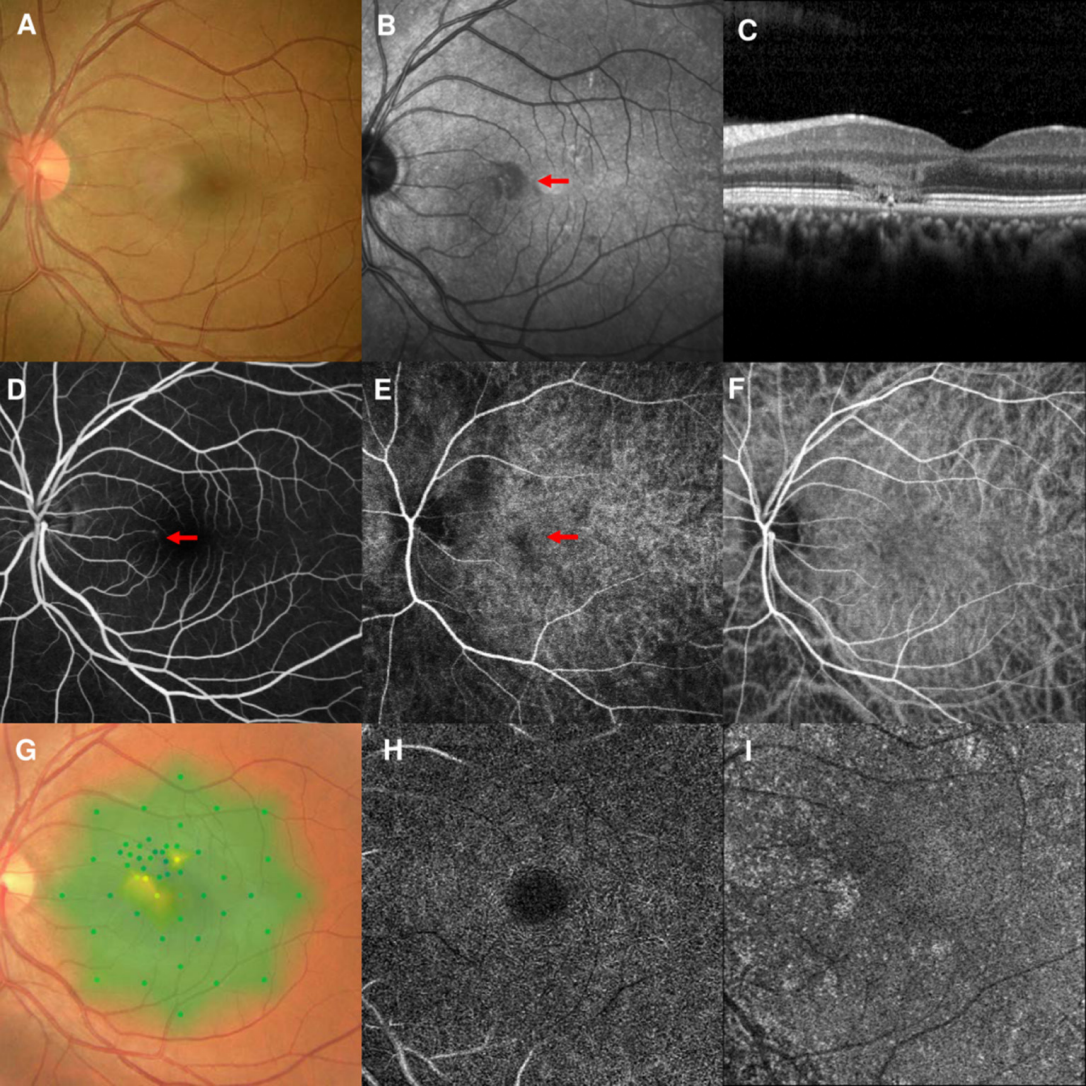
Case 2: Multimodal imaging of acute macular neuroretinopathy in the left eye. Fundus photography **(A)** and corresponding near-infrared reflectance imaging (NIR) demonstrated a hyporeflective lesion at the nasal part of the macula in the left eye [**(B)**, red arrowhead]. Spectral-domain optical coherence tomography (OCT) line scans showed hyperreflectivity at the level of the outer plexiform and outer nuclear layers and attenuation of the ellipsoid zone **(C)**. The middle phase of fluorescein angiography (FA) showed dot leakage at the end of nasal macular arterioles **(D)**. The early phase of indocyanine green angiography (ICGA) showed a patchy choroidal filling area [**(E)**, arrowheads], which could not be observed after 2 s **(F)**. Microperimetry revealed scotomata corresponding to the hyporeflective lesions on NIR **(G)**. OCT angiography showed normal vascular flow at the deep capillary plexus **(H)** and choriocapillaris **(I)**.

A 33-year-old man had a 2-day history of a central scotoma in the left eye. He had undergone LASIK surgery 12 years earlier. The patient denied any medication use, recent vaccinations, or other illnesses. The BCVA was 20/30 OS. The fundus examination showed edema at the nasal part of the macula in the left eye (Figure 2A). NIR showed a hyporeflective area that corresponded to the edema detected on fundus examination (Figure 2B). SD-OCT showed profound OPL and ONL hyperreflectivity and attenuation of the ellipsoid zone (Figure 2C). The arteriovenous phase of the FA showed dot leakage at the terminus of the nasal macular arterioles (Figure 2D, marked by red arrowhead). Early-phase ICGA showed a hypofluorescent area considered to be PCF at the nasal part of the macula, which was associated with the AMN lesions (Figure 2E). This PCF only lasted 2 s on ICGA (Figure 2F). FA and ICGA showed that the PCF was at the terminus of the macular arterioles. Microperimetry revealed decreased retinal sensitivity corresponding to the hyporeflective lesions on NIR (Figure 2G). OCT-A could not identify the vascular flow voids at the level of the DCP (Figure 2H) or CC (Figure 2I). The patient's vision improved to 20/20 OS after 6 months of follow-up.

### Case 4

**Figure 3:**
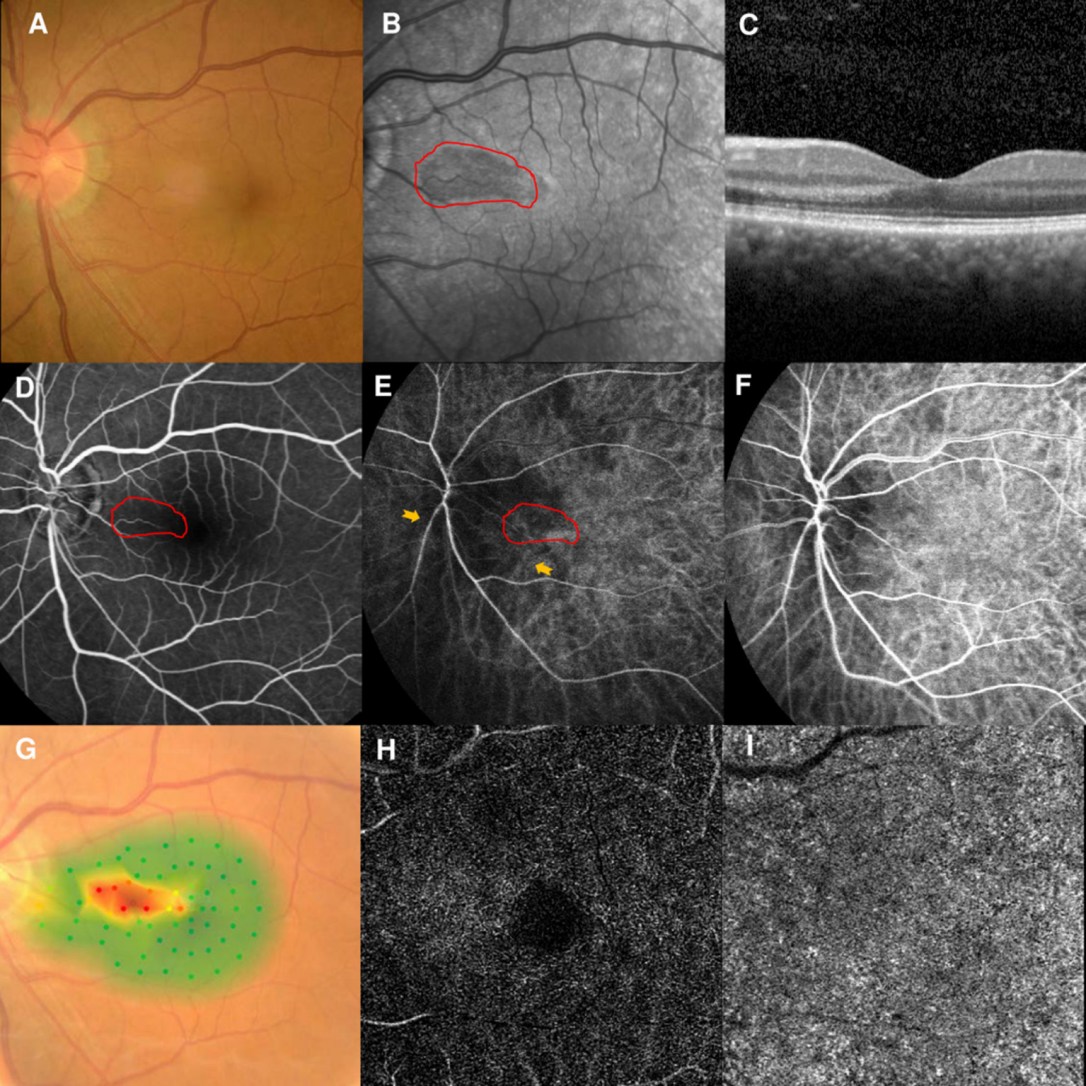
Case 4: Multimodal imaging of acute macular neuroretinopathy in the left eye. Fundus photography **(A)** and corresponding near-infrared reflectance imaging (NIR) demonstrated a hyporeflective lesion at the nasal part of the macula in the left eye [**(B)**, red line]. Spectral-domain optical coherence tomography (OCT) through the lesion showed hyperreflectivity at the outer plexiform and outer nuclear layers **(C)**. The findings for the middle phase of fluorescein angiography (FA) were unremarkable in the left eyes, and a macular venule passing through the AMN lesion was observed **(D)**. Indocyanine green angiography (ICGA) showed a vertical watershed zone coursing through the optic disc and extending toward the fovea [**(E)**, between two yellow arrowheads]. The AMN lesion was at the border of the watershed zone [**(E)**, red line], and this watershed zone could not be observed after 4 s **(F)**. The corresponding hypofluorescence areas on NIR were identified and colocalized; these are marked by the red line, with the noted areas on FA and ICGA. Microperimetry revealed scotomata corresponding to the hyporeflective lesions on NIR **(G)**. OCT angiography showed normal vascular flow at the deep capillary plexus **(H)** and choriocapillaris **(I)**.

A 56-year-old man had a 1-week history of blurred vision in the left eye. He had undergone partial nephrotomy due to renal hamartomas 2 years earlier, but denied any other illnesses and medication use. His BCVA was 20/40 and fundus examination showed perifoveal edema in the left eye (Figure 3A). NIR of the left eye showed a tear-drop hyporeflective lesion pointing toward the fovea (Figure 3B). SD-OCT showed hyperreflectivity of the OPL and ONL (Figure 3C). FA showed a macular venule passing through the AMN lesion (Figure 3D), and early-stage ICGA could identify a vertical watershed zone coursing through the optic disc and extending toward the fovea (Figure 3E, between two yellow arrowheads). The AMN lesion was at the border of the watershed zone; the watershed zone could not be observed on ICGA after 4 s (Figure 3F). Microperimetry revealed a scotoma corresponding to the hyporeflective lesions on NIR (Figure 3G). OCT-A showed normal vascular flow at the level of the DCP (Figure 3H) and CC (Figure 3I). The patient's vision improved to 20/30 OS after 10 months of follow-up, however, the paracentral scotoma of the left eye persisted.

### Case 5

**Figure 4:**
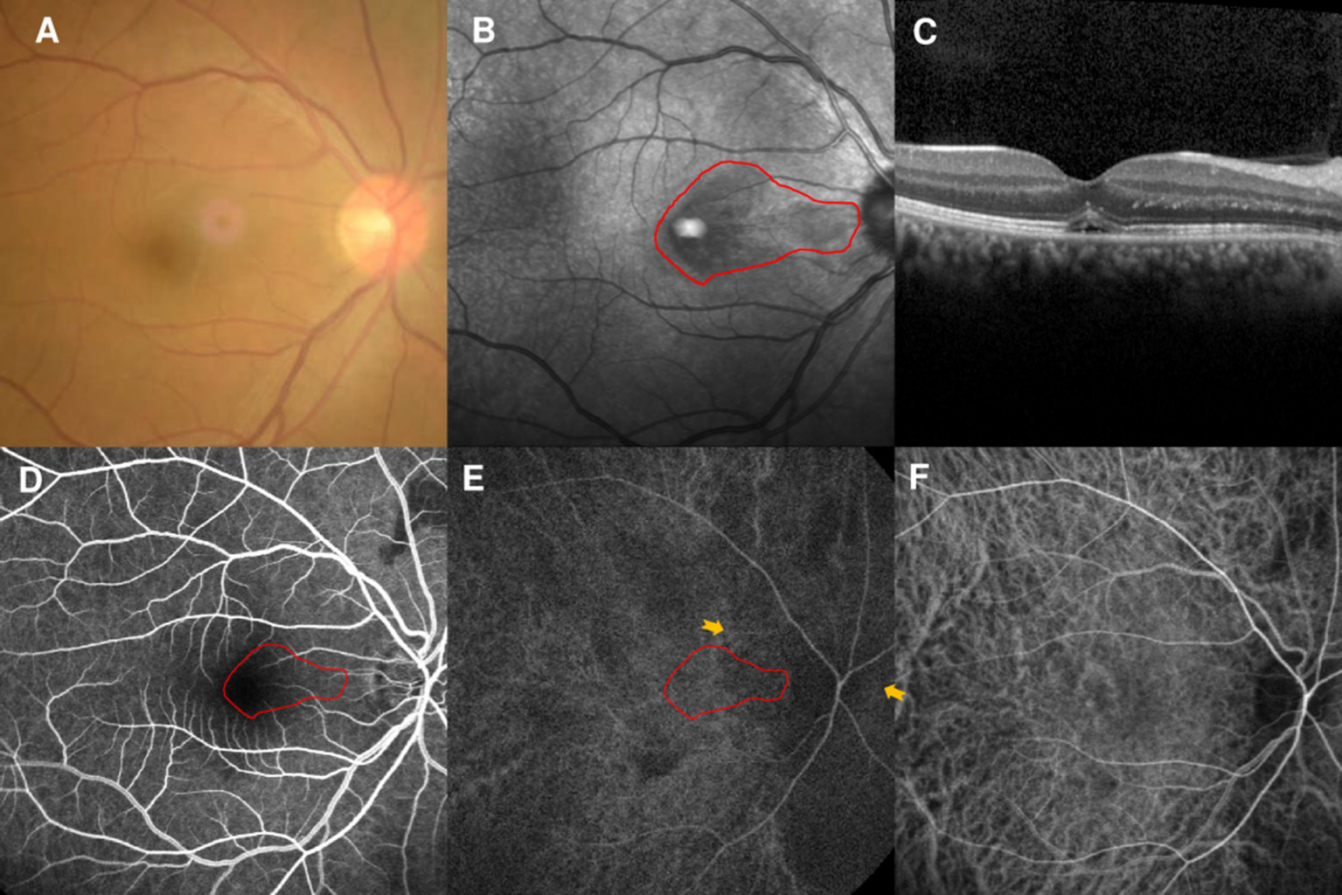
Case 5: Multimodal imaging of acute macular neuroretinopathy in the right eye. Fundus photography **(A)** and corresponding near-infrared reflectance imaging (NIR) demonstrated a hyporeflective lesion at the nasal part of the macula in the left eye [**(B)**, red line]. Spectral-domain optical coherence tomography (OCT) through the lesion showed hyperreflectivity at the outer plexiform and outer nuclear layers and sub-retinal fluid under the fovea **(C)**. The arteriovenous phase of fluorescein angiography (FA) of the right eye showed unremarkable findings **(D)**. Indocyanine green angiography (ICGA) showed a vertical watershed zone coursing through the optic disc [**(E)**, between two yellow arrowheads]. The AMN lesion was crossing the watershed zone [**(E)**, red line], and this watershed zone could not be observed after 8 s **(F)**. The corresponding hypofluorescent areas detected by NIR were identified and colocalized; these are marked by the red line, with the noted areas on FA and ICGA.

A 37-year-old man first visited our clinic in August 2020, presenting a 1-week history of scotoma in the right eye. He had mild myopia and hypertension, but denied any medication use, recent vaccinations, or other illnesses. The BCVA was 20/40 OD and fundus examination findings were unremarkable (Figure 4A). NIR showed multiple irregular hyporeflective areas in the right eye (Figure 4B). SD-OCT showed OPL and ONL hyperreflectivity and tiny sub-retinal fluids under the fovea (Figure 4C). FA showed a normal circulation (Figure 4D). Early-stage ICGA detected a vertical watershed zone coursing through the optic disc; the AMN lesions crossed the watershed zone (Figure 4E). This watershed zone lasted 8 s on ICGA (Figure 4F). After a negative finding on general check-up, observation was recommended.

**Figure 5:**
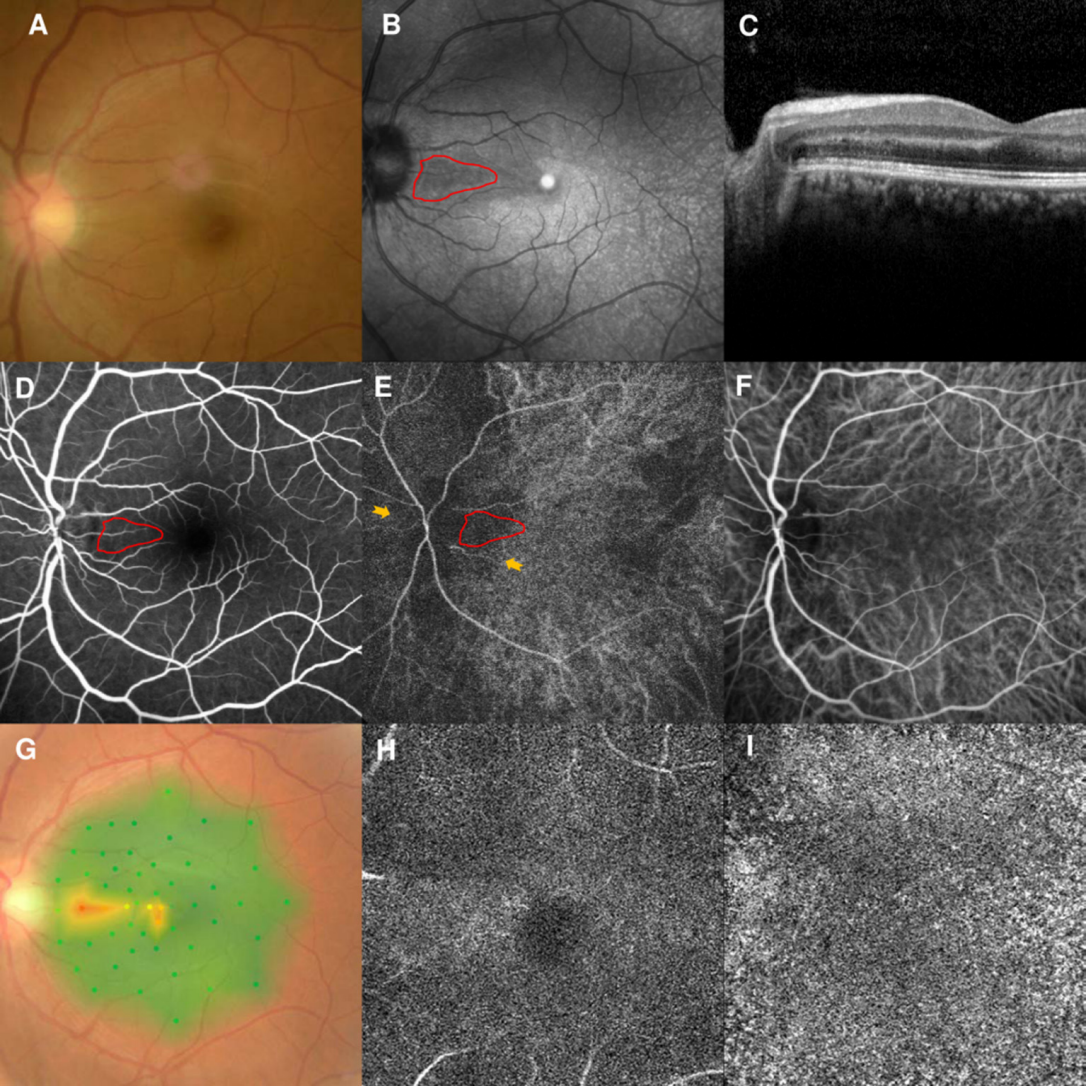
Case 5: Multimodal imaging of acute macular neuroretinopathy in the left eye. Fundus photography **(A)** and corresponding near-infrared reflectance imaging (NIR) demonstrated a hyporeflective lesion at the nasal part of the macula in the left eye [**(B)**, red line]. Spectral-domain optical coherence tomography (OCT) through the lesion showed hyperreflectivity at the outer plexiform and outer nuclear layers **(C)**. The arteriovenous phase of fluorescein angiography (FA) showed unremarkable findings for the left eyes **(D)**. Indocyanine green angiography (ICGA) showed a vertical watershed zone coursing through the optic disc [**(E)**, between two yellow arrowheads]. The AMN lesion was situated at the watershed zone [**(E)**, red line], and this watershed zone could not be observed after 5 s **(F)**. The corresponding hypofluorescence areas on NIR were identified and colocalized; these are marked by the red line, with the noted areas on FA and ICGA. Microperimetry revealed a scotoma corresponding to the hyporeflective lesions on NIR **(G)** OCT-A could not detect the vascular flow voids at the level of the DCP **(H)** or CC **(I)**.

However, the patient returned to our clinic in February 2021 and complained of similar symptoms in the left eye for 2 days. His BCVAs were 20/25 OD and 20/30 OS, and fundus examination findings were unremarkable (Figure 5A). NIR of the left eye showed a tear-drop hyporeflective lesion (Figure 5B). SD-OCT showed OPL and ONL hyperreflectivity (Figure 5C). FA showed normal circulation (Figure 5D). Early-stage ICGA detected a vertical watershed zone; the AMN lesions were located within the watershed zone (Figure 5E). This watershed zone lasted 5 s on ICGA (Figure 5F). Microperimetry revealed a scotoma corresponding to the hyporeflective lesions on NIR (Figure 5G) OCT-A could not detect the vascular flow voids at the level of the DCP (Figure 5H) or CC (Figure 5I). The vision improved to 20/25 in both eyes after 6 months of follow-up.

### Case 6

**Figure 6:**
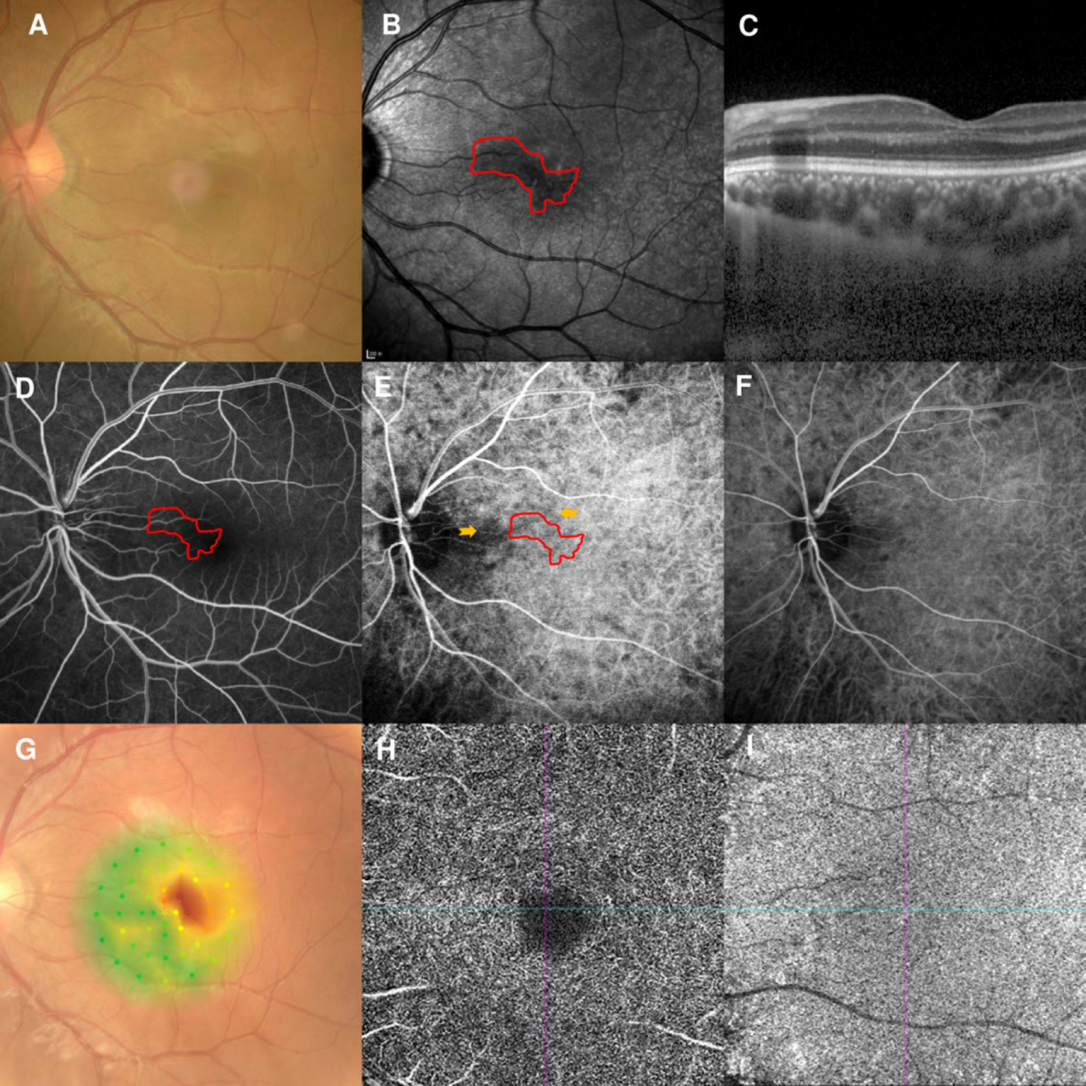
Case 6: Multimodal imaging of acute macular neuroretinopathy of the left eye. Fundus photography **(A)** and corresponding near-infrared reflectance imaging (NIR) demonstrated an irregular hyporeflective lesion at the nasal part of the macula in the left eye [**(B)**, red line]. Spectral-domain optical coherence tomography (OCT) through the lesion showed hyperreflectivity at the outer plexiform layer **(C)**. The arteriovenous phase of fluorescein angiography (FA) showed unremarkable findings **(D)**. Indocyanine green angiography (ICGA) showed a vertical watershed zone coursing through the optic disc [**(E)**, between two yellow arrowheads]. The AMN lesion was situated at the watershed zone [**(E)**, red line], and this watershed zone could not be observed after 4 s **(F)**. The corresponding hypofluorescent areas on NIR were identified and colocalized; these were marked by the red line, with the noted areas on FA and ICGA. Microperimetry revealed a scotoma corresponding to the hyporeflective lesions on NIR **(G)** OCT-A could not detect the vascular flow voids at the level of the DCP **(H)** or CC **(I)**.

A 20-year-old man had a 2-week history of blurred vision and a central scotoma in the left eye. He was diagnosed with optic neuritis in another hospital and sought a second opinion at our clinic. The patient denied any medication use, recent vaccinations, or other illnesses. He also denied orbital pain before and while he had his vision symptoms. The BCVA was 20/200 OS, and there was no relative afferent pupillary defect in the left eye. The fundus examination findings were unremarkable (Figure 6A). NIR showed irregular hyporeflective areas in the left eye (Figure 6B). SD-OCT showed OPL hyperreflectivity (Figure 6C), while the FA showed normal circulation (Figure 6D). Early-stage ICGA detected an irregular watershed zone and a narrow branch coursing from the optic disc to the macula; the AMN lesions crossed at this narrow branch (Figure 6E). This watershed zone lasted 4 s on ICGA (Figure 6F). Microperimetry revealed an irregular scotoma (Figure 6G). OCT-A could not detect the vascular flow voids at the level of the DCP (Figure 6H) or CC (Figure 6I). The brain and orbital magnetic resonance imaging and enhancement findings were within normal limits. Antibodies targeting aquaporin-4 and myelin oligodendrocyte glycoprotein were also negative. AMN was diagnosed, and observation was recommended. The patient's vision improved to 20/50 after 3 months of follow-up.

## Discussion

Ischemia has been well-accepted as the etiology of AMN despite some controversies. This has also been supported by a recent study showing the coexistence of AMN and other ischemic retinal diseases (8). Previous research has shown that the OPL demonstrates the earliest involvement in AMN (3). As the DCP is within the inner border of the OPL, local ischemia of the DCP has been suggested in the pathogenesis of AMN (3). With the development of the OCT-A technique, some studies have found that CC flow voids co-localized with the lesions detected on NIR, suggesting vascular impairment of the CC, and not the DCP, as the possible cause of AMN (9, 10). However, these results are controversial. Some authors have questioned whether the flow void signal within the CC observed on OCT-A is due to an artifact caused by a flow signal blockage from the hyper-reflectance of the overlying outer retina (11), and other OCT-A studies have shown that AMN lesions were associated with a reduced signal in the DCP only (12) or both the DCP and CC (13). We could not identify the flow void within the DCP or CC on OCT-A in our cases. These conflicting results may represent a limitation of the consistency of the different techniques and algorithms used for OCT-A (14). The origin and location of the vascular impairment of AMN have not been established.

Our study showed that AMN lesions have a topographical relationship with the CWZ or PCF. The CWZ indicates the isolation of the choriocapillaris bed supplied by independent posterior ciliary arteries (PCA) that do not anastomose with each other. The CWZ plays a pathological role in several diseases because of its relatively low blood flow (4, 15). Recent studies have shown that idiopathic CNV, AMD, and polypoidal choroidal vasculopathy have topographical relationships with the CWZ (6, 7). PCF (or delay choroidal filling) indicates a well-defined hypofluorescent region during the early stage of ICGA or FA, which usually has an irregular shape and may be isolated from the CWZ. PCF is the most common form of choroidal vascular filling defect (16). PCF has several similar characteristics to the CWZ. First, PCF shows early hypofluorescence and normal filling on FA or ICGA (16). Second, previous studies have shown that PCF also has a topographical relationship with AMD and pathological myopia CNV (5, 17). Therefore, some researchers speculate that PCF may partially originate from the CWZ (17).

Previous studies have shown that the CWZ and PCF have a pathological role because of their long-term reduced blood flow, but AMN is considered an acute process. Our study found that the AMN lesions were located within the retinal area with a relatively lower oxygen supply, in addition to having a topographical relationship with the CWZ and PCF. The lesions of cases 1, 2, 6, and the right eye of case 5 were at the terminals of the perifoveal vessels (Figures 1E, 2D, 4D, 6D), and the lesions of case 4 and the left eye of case 5 were at the border of the perifoveal venule (Figures 3D, 4D). These features indicate that the retinal area involved had the poorest oxygen supply. The retina has a dual blood supply, and the avascular nature of the outer retina makes it vulnerable to acute changes in oxygen supply or demand (18). Moreover, the OPL, which has the earliest involvement in AMN, has a relatively high oxygen consumption due to its greater synaptic activity (18). Thus, this retinal area was most vulnerable to acute hypoperfusion.

The outer retina is mostly supplied by the choroidal vascular bed, but it still has some oxygen supply from the DCP. There is no clear boundary for the layer supplied by the retinal or choroidal circulation (18). Yu and Cringle (19) demonstrated that the oxygen flux significantly increased from the DCP to the photoreceptors during dark adaptation. Thus, the oxygen supply of the OPL, which is located close to the DCP, may be derived from a delicate balance between the retinal and choroidal circulation. Based on this evidence, it is difficult to attribute AMN solely to ischemia of the CC or DCP.

**Figure 7:**
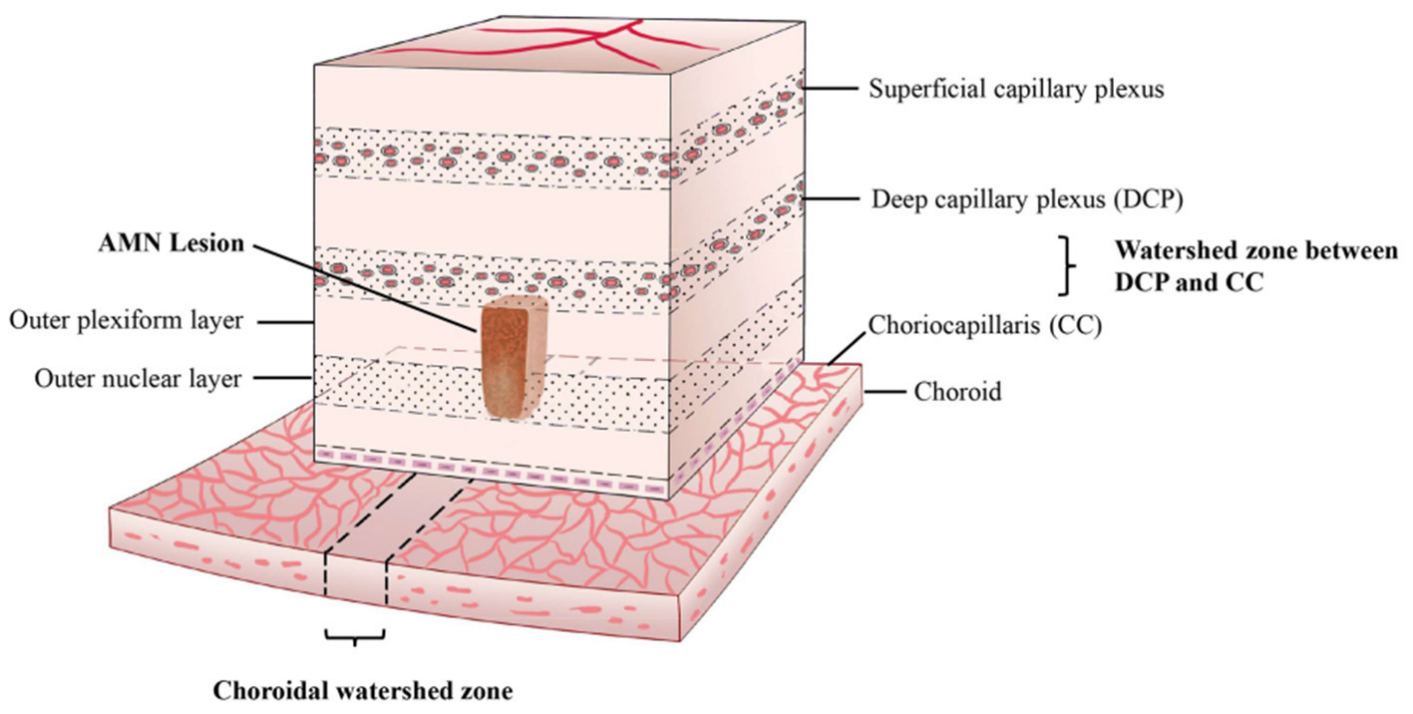
Proposed three-dimensional illustration of the dual watershed zone hypothesis. The AMN lesions are within the watershed zone between the deep capillary plexus and choriocapillaris and above the watershed zone of the choroid. This characteristic dual watershed zone makes this retinal area most vulnerable to hypoperfusion.

We speculated that the pathophysiological mechanism of AMN is similar to the “watershed infarction” of the brain. Watershed infarcts are ischemic lesions involving the junction of the distal fields of two non-anastomosing arterial systems (20). From our study, we showed that AMN lesions were not only at the functional anteroposterior watershed between the DCP and CC; they also had a topographical relationship with the CWZ or PCF. This characteristic “dual watershed zone” made this area most vulnerable to hypoperfusion events. The hypoperfusion may be at the level of the ophthalmic artery or above, which was speculated in a previous study (21), and can lead to reduced perfusion of the dual vessel system. Transient hypoperfusion may cause watershed infarction of this area of the retina. In contrast with focal infarction, watershed infarction is not caused by a total occlusion of a local end-vessel (22); this may explain why AMN lesions typically had no FA or ICGA correlates in previous studies and had no signal voids on OCT-A in our study. A schematic of our understanding of the watershed zone and AMN lesion is shown in Figure 7.

This hypothesis may explain several clinical observations. First, paracentral acute middle maculopathy (PAMM) shares several similar clinical features with AMN and was initially thought to be a variant of AMN (23). Several studies demonstrate that vascular flow voids in the DCP correlate with PAMM lesions (24). In addition, numerous studies have shown that PAMM has a close relationship with other retinal ischemic diseases, such as retinal vein occlusion and artery occlusion (8, 25, 26). This raises the following question: if DCP ischemia is the etiology of AMN (as it is for PAMM), why does AMN occur less frequently in association with other retinal vascular diseases than PAMM? From our hypothesis, this may be attributed to PAMM requiring only retinal circulation hypoperfusion, while AMN requires hypoperfusion of both retinal and choroidal blood sources. Second, AMN lesions were likely to be located at the nasal part of the macula in previous cases (1, 2). This may be caused by the location of the CWZ. In a recent dynamic indocyanine green study, Cheung et al. (27) demonstrated that the fluorescence dye traveled radially from the peripapillary region to the macular region and finally the peripheral area. This was caused by the emergence of short PCAs from the lateral or medial PCA (4), and most of the watershed zone was situated within the optic disc and sometimes extended to the macular area (28, 29).

A major limitation of the current study is the small sample size and its retrospective nature, which can be attributed to the rareness of AMN. Another study limitation is that only case 4 underwent videoangiography, which can more accurately distinguish CWZ or PCF. It should also be noted that case 3 showed no topographical relationship between AMN and CWS or PCF (Supplementary Figure 1). Case 3 is the only case with a clear flu-like prodrome in our study. A flu-like prodrome was reported to have a 47.5% association with AMN (2); another study showed that AMN is a major manifestation of dengue maculopathy (30). With the outbreak of coronavirus disease 2019 (COVID-19), the coexistence of AMN and COVID-19 has been reported more frequently (31, 32). These findings raise the question of whether the etiology of virus-related AMNs is ischemia-induced outer retina edema, or virus-induced outer retina inflammation? Different cases may have different etiologies (inflammation or ischemia), and our theory can only explain ischemia induced AMN lesions. Finally, the mechanisms proposed by this study need to be supported by histopathological investigations in the future.

## Conclusions

In summary, our study demonstrates that AMN lesions have topographical relationships with the CWZ or PCF based on multimodal imaging findings. Furthermore, we showed that the retinal locations of AMN lesions are the most vulnerable to acute hypoperfusion. We used the “dual-watershed zone” to explain the etiology of ischemia-induced AMN lesions. Our study sheds new light on the pathophysiological mechanisms of this rare disease. We acknowledge that this case series is small, and a cause-and-effect association cannot be determined. Further studies are needed to validate our hypothesis.

## Data Availability Statement

The raw data supporting the conclusions of this article will be made available by the authors, without undue reservation.

## Ethics Statement

The studies involving human participants were reviewed and approved by Ethical Committee of the Second Hospital of Hebei Medical University. The patients/participants provided their written informed consent to participate in this study.

## Author Contributions

JD, JA, JM, and QS conceived and designed the study. JA, JM, and QS recruited the patients. ML, PY, and LZ collected the data. JD and JA performed the literature search and the interpretation of data. JD and QS wrote the manuscript. JM, ZZ, and LZ made a critical revision of the article. All authors approved the final manuscript.

## Funding

This study was supported by the Natural Science Foundation of Hebei Province (Grant No. H2020206063). Top Talent Support Program for Young and Middle-Aged People of the Wuxi Health Committee (Grant No. HB2020030).

## Conflict of Interest

The authors declare that the research was conducted in the absence of any commercial or financial relationships that could be construed as a potential conflict of interest.

## Publisher's Note

All claims expressed in this article are solely those of the authors and do not necessarily represent those of their affiliated organizations, or those of the publisher, the editors and the reviewers. Any product that may be evaluated in this article, or claim that may be made by its manufacturer, is not guaranteed or endorsed by the publisher.
