# Supplementary figures and images for: Topographical Relationship Between Acute Macular Neuroretinopathy and Choroidal Watershed Zone or Patchy Choroidal Filling
Source: Front Med (Lausanne). 2022 Feb 1;9:762609. doi: 10.3389/fmed.2022.762609 (PMC8843832; doi:10.3389/fmed.2022.762609)

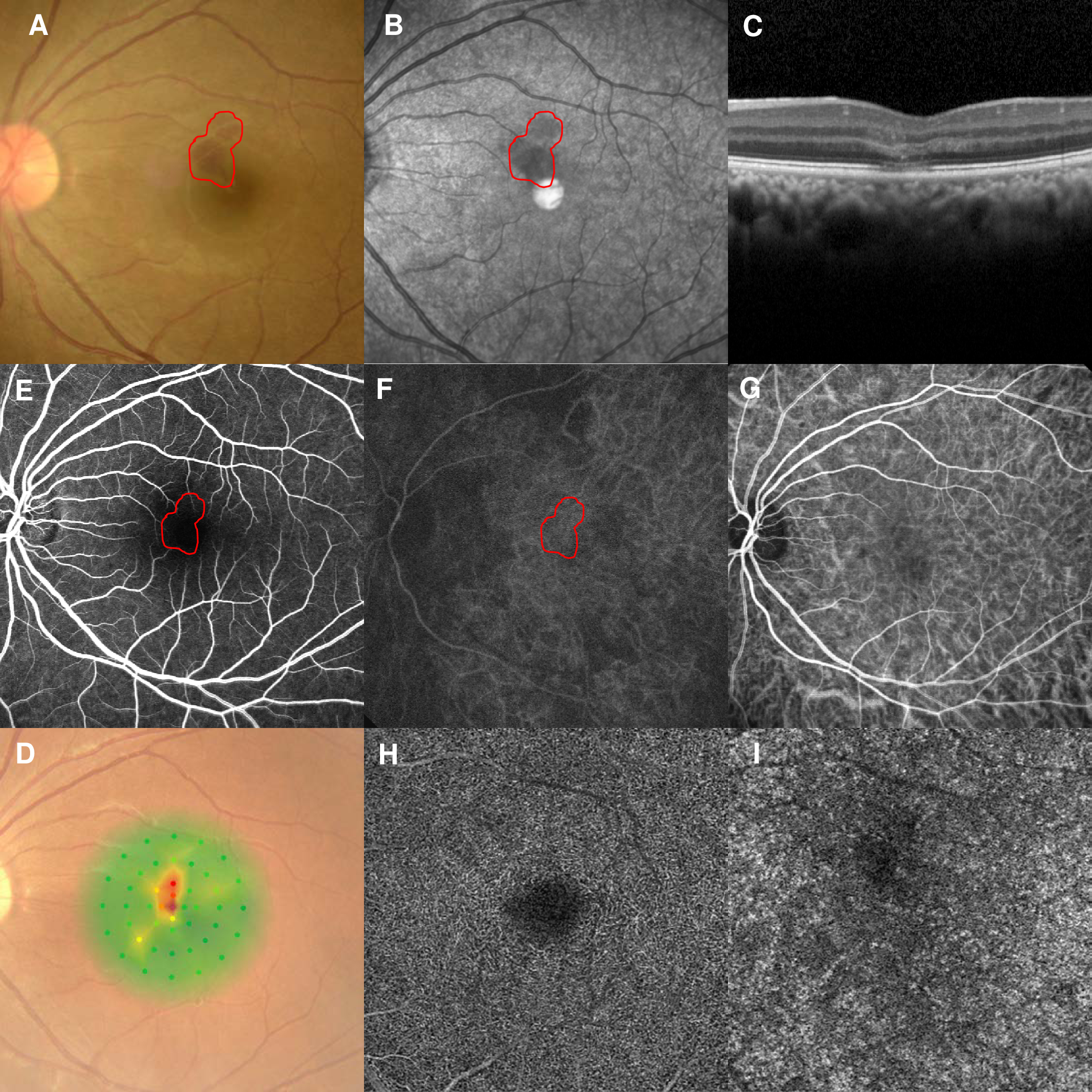

Supplement: Supplementary file 1 [file Image_1.TIF]
